# Development and validation of a nomogram to provide individualized predictions of functional outcomes in patients with convulsive status epilepticus at 3 months: The modified END‐IT tool

**DOI:** 10.1111/cns.14313

**Published:** 2023-06-19

**Authors:** Xuan Wang, Yuan‐Yuan Wang, Qiong Gao, Yao‐Yao Zhang, Jian Wan, Chang‐Geng Song, Jing‐Ya Wei, Xiao‐Gang Kang, Fang Yang, Wen Jiang

**Affiliations:** ^1^ Department of Neurology Xijing Hospital, Fourth Military Medical University Xi'an China; ^2^ State Key Laboratory of Cancer Biology Xijing Hospital of Digestive Diseases, Fourth Military Medical University Xi'an China

**Keywords:** convulsive status epilepticus, functional outcomes, modified END‐IT, prediction model

## Abstract

**Aims:**

The prediction of outcomes in convulsive status epilepticus (CSE) remains a constant challenge. The Encephalitis‐Nonconvulsive Status Epilepticus‐Diazepam Resistance‐Image Abnormalities‐Tracheal Intubation (END‐IT) score was a useful tool for predicting the functional outcomes of CSE patients, excluding cerebral hypoxia patients. With further understanding of CSE, and in view of the deficiencies of END‐IT itself, we consider it necessary to modify the prediction tool.

**Methods:**

The prediction model was designed from a cohort of CSE patients from Xijing Hospital (China), between 2008 and 2020. The enrolled subjects were randomly divided into training cohort and validation cohort as a ratio of 2:1. The logistic regression analysis was performed to identify the predictors and construct the nomogram. The performance of the nomogram was assessed by calculating the concordance index, and creating calibration plots to check the consistency between the predicted probabilities of poor prognosis and the actual outcomes of CSE.

**Results:**

The training cohort included 131 patients and validation cohort included 66 patients. Variables included in the nomogram were age, etiology of CSE, non‐convulsive SE, mechanical ventilation, abnormal albumin level at CSE onset. The concordance index of the nomogram in the training and validation cohorts was 0.853 (95% CI, 0.787–0.920) and 0.806 (95% CI, 0.683–0.923), respectively. The calibration plots showed an adequate consistency between the reported and predicted unfavorable outcomes of patients with CSE at 3 months after discharge.

**Conclusions:**

A nomogram for predicting the individualized risks of poor functional outcomes in CSE was constructed and validated, which has been an important modification of END‐IT score.

## Graphical abstract

A nomogram prediction of functional outcome in patients with convulsive status epilepticus.

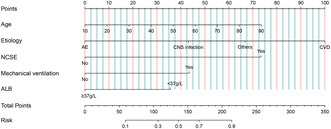

## 1 BACKGROUND

Convulsive status epilepticus (CSE) is a common neurological emergency and is characterized by significant mortality.^1^, ^2^ Even if some patients survive CSE, they subsequently will still develop neurological deficits. During clinical practice, a clinical tool to help predict the individualized risks for poor functional outcomes in CSE patients is useful, especially when clinicians optimize treatment strategies and communicate with relatives.

The Encephalitis‐Nonconvulsive Status Epilepticus‐Diazepam Resistance‐Image Abnormalities‐Tracheal Intubation (END‐IT) score, which was proposed in our previous work, was the only clinical tool used to predict the functional outcomes of patients with CSE after discharge.^3^ It is a comprehensively simple and practical prediction tool. In recent years, the predictive value of END‐IT has been validated in some medical centers.^4^, ^5^, ^6^ Some researchers supported the use of END‐IT, especially in patients under 65 years old,^4^ while other studies found it might not be a satisfactory prediction tool to some extent.^5^, ^6^ The under‐categorization of etiologies of CSE was noted and the item of image was questioned.^5^, ^6^ With progressing research and further understanding of status epilepticus (SE), we found that the END‐IT score system does have some deficiencies, which need to be modified now.

Hence, in this prognostic study, we reassessed the existing variables in END‐IT score system, as well as other recently identified risk factors for poor outcomes in CSE, based on a larger case database. The aim was to modify and validate the prediction model for estimating the risk of poor functional outcomes post‐discharge in CSE. In addition, compared with the scoring system, nomograms could provide a more individualized prediction since continuous predictors do not need to be categorized, and the website calculates have interactive graphical interfaces and are very convenient to use.^7^ Here, a graphical nomogram and a web‐based tool were developed to present the modified model in a visualized and easy‐to‐access manner.

## 2 MATERIALS AND METHODS

### 2.1 Study setting, patients, and treatments

This study was carried out based on a prospective registry of consecutive patients with CSE in the neurological intensive care unit (neuro‐ICU), Department of Neurology, Xijing Hospital, from March 2008 to June 2020. Informed consent was obtained from the patients or their guardians. The procedures of this study were approved by the ethics committee of Xijing Hospital, Fourth Military Medical University (KY20182024‐F‐1) and adhered to the Helsinki Declaration. The study followed the Transparent Reporting of a Multivariable Prediction Model for Individual Prognosis of Diagnosis (TRIPOD) reporting guideline for prognostic studies.

Patients who were older than 12 years and fulfilled the International League Against Epilepsy's definition of CSE were enrolled for this study.^8^ Almost all patients with SE from cerebral hypoxia always had a dismal prognosis, therefore, these patients were excluded. Patients discharged from the hospital against the advice of doctors and patients whose data were incomplete were also excluded from this study. After admission, each patient underwent a detailed history‐taking and physical examination. Experienced specialists in neuro‐intensive care developed the treatment regimen. Every patient was monitored continuously using bedside video electroencephalography (EEG) with an array of 16 scalp electrodes (Solar 2000 N; Solar Electronic Technologies Co, Ltd.) for seizure detection and efficacy assessment of anti‐seizure treatment. Rapid termination of epileptic activity is the main goal of treatment. All the patients received anti‐SE treatments according to the Chinese expert consensus which agrees with the American Epilepsy Society Guideline.^9^, ^10^ In the initial therapy phase, intravenous (IV) diazepam was administered as the first‐line option. Diazepam resistance was defined as no response to one repeated IV full dose of diazepam. When the seizure duration reached 20 min, the second‐therapy phase began and options included IV valproic acid, levetiracetam, or phenobarbital. If the initial and second therapies failed and the seizure duration reached 40 min, anesthetic doses of either midazolam, propofol, or phenobarbital were given to patients. At the same time, patients also received necessary life support and organ protection. Physicians monitored closely for potential serious complications in therapeutic coma, including cardiotoxicity from phenobarbitone, and hepatotoxicity and metabolic acidosis with rhabdomyolysis and cardiac failure (propofol infusion syndrome) from propofol. Treatment of the underlying critical illness and additional general care continued throughout. All cases were treated carefully and received close clinical follow‐up. Refractory status epilepticus (RSE) was defined as seizures that persisted after therapy with two anti‐seizure drugs, typically including a benzodiazepine. Super‐RSE (S‐RSE) was defined as SE that continued for 24 h or more despite anesthetic treatment or recurred on attempted weaning of the anesthetic regimen.^11^, ^12^ NCSE was defined as a continuous state of seizures without convulsions or multiple nonconvulsive seizures more than 30 min without interictal full recovery and was validated by the Salzburg criteria.^13^, ^14^, ^15^

### 2.2 Data collection

The demographic data and clinical parameters were collected from the electronic medical records system, including age, sex, CSE etiologies, neuroimaging findings, anti‐SE treatments, complications, and albumin (ALB) at CSE onset. The underlying etiology of CSE was categorized according to the ‘List of Etiologies That May Cause Status Epilepticus’ provided for clinicians by International League Against Epilepsy (IALE) in 2015.^8^ All the brain computed tomography (CT), magnetic resonance imaging (MRI) scans and EEG results of each patient were analyzed by experienced radiologists and neurologists together for the identification of brain regions generating SE. The brain lesions were classified into three different categories: no responsible lesion, unilateral responsible lesions, and bilateral responsible lesions or diffuse cerebral edema. The clinical outcome was routinely evaluated 3 months after discharge through telephone by a trained neurologist who was blinded to the clinical data. Modified Rankin Scale (mRS) score^16^ was used to assess the disease outcome. An mRS score of 0–2 indicated a good outcome and an mRS score of 3–6 indicated a poor outcome.

### 2.3 Statistical analysis

Based on our clinical experience and from a search of published studies,^2^, ^17^, ^18^, ^19^, ^20^ we identified the following variables as probable predictors for the poor functional outcome in CSE. These were age, sex, etiologies of CSE, image findings, NCSE, diazepam resistance, the use of anesthetics, mechanical ventilation and abnormal ALB at the onset of CSE (37 g/L is the threshold that distinguishes normal albumin levels in our hospital). Continuous data were presented as the mean ± standard deviation (normally distributed) or median (interquartile range [IQR], not normally distributed). Shapiro–Wilk formal test was used to assess data distribution. Continuous variables were analyzed using Student's *t*‐test (normally distributed) or Mann–Whitney *U*‐test (not normally distributed). Categorical variables were expressed as counts and percentages. Categorical variables were analyzed using the *χ* ^2^ test or Fisher's exact test if any expected value was below five.

The subjects were randomly divided into training cohort and validation cohort as a ratio of 2:1 in order to construct and validate the nomogram. Univariate logistic analysis was carried out to identify the risk factors for the poor prognosis of CSE patients in the training cohort. The significant factors (*p* < 0.10) in the univariate analysis were included in the multivariate logistic analysis. Following the multivariate logistic analysis, factors with *p* < 0.05 were considered as the independent predictors and used to develop the predictive nomogram. The discrimination of the nomogram was quantified by the concordance index (C‐index) with a 95% confidence interval (95% CI). The value of the area under the receiver operating characteristic (ROC) curve was similar to the C‐index, with 0.5 representing random chance and 1.0 representing correct predictions for all patients.^21^ Calibration plots were created to check the consistency between the actual outcomes and predicted probabilities of poor prognosis of SE patients at 3 months after discharge. Statistical analysis was performed using the R software, version 4.0.5 (R Foundation for Statistical Computing). A two‐tailed *p*‐value <0.05 was considered statistically significant.

## 3 RESULTS

A total of 197 patients with CSE were enrolled in the final analysis of this prognostic study. Among them, 131 patients were included in the training group and 66 patients were included in the validation group (Figure S1).

### 3.1 Characteristics and outcomes of patients

The characteristics of patients with CSE were shown in Table 1. Among all patients, the mean age was 36.6 ± 18.9 years. 103 patients (52.3%) were male and 94 patients (47.7%) were female. The top three etiologies of CSE were autoimmune encephalitis (24.9%), central nervous system (CNS) infection (23.9%) and cerebral vascular disease (14.7%). 45.2% (89/197) of the CSE patients got a poor outcome at 3 months after discharge, while the other 54.8% (108/197) of the patients got a satisfied prognosis. There was no significant difference in age, sex, etiologies of CSE, neuroimaging findings, the incidence of RSE/S‐RSE and NCSE, diazepam resistance rate, the utilization of anesthetics, the rate of mechanical ventilation, the proportion of patients whose serum ALB <37 g/L, and the outcomes at 3 months post‐discharge between the training and validation cohorts.

**TABLE 1:** Baseline characteristics of patients in the training and validation cohorts.

Variables	Overall (*n* = 197)	Training cohort (*n* = 131)	Validation cohort (*n* = 66)	*p* Value
Age, mean (SD)	36.6 (18.9)	37.1 (19.5)	35.7 (17.6)	0.625
Male, *n* (%)	103 (52.3)	67 (51.1)	36 (54.5)	0.764
Etiology, *n* (%)				
AE	49 (24.9)	36 (27.5)	13 (19.7)	0.397
CNS infection	47 (23.9)	30 (22.9)	17 (25.8)	
CVD	29 (14.7)	16 (12.2)	13 (19.7)	
Others	72 (36.5)	49 (37.4)	23 (34.8)	
Image, *n* (%)				
Normal	60 (30.5)	40 (30.5)	20 (30.3)	0.514
Unilateral lesions	80 (40.6)	50 (38.2)	30 (45.5)	
Bilateral lesions/diffuse cerebral edema	57 (28.9)	41 (31.3)	16 (24.2)	
RSE/S‐RSE	82 (41.6)	54 (41.2)	28 (42.5)	0.872
NCSE, *n* (%)	69 (35.0)	49 (37.4)	20 (30.3)	0.408
Diazepam resistance, *n* (%)	116 (58.9)	81 (61.8)	35 (53.0)	0.302
Anesthetics, *n* (%)	68 (34.5)	46 (35.1)	22 (33.3)	0.929
Mechanical ventilation, *n* (%)	81 (41.1)	57 (43.5)	24 (36.4)	0.419
ALB < 37 g/L, *n* (%)	76 (38.6)	52 (39.7)	24 (36.4)	0.765
Outcome, *n* (%)				
Good	108 (54.8)	69 (52.7)	39 (59.1)	0.482
Poor	89 (45.2)	62 (47.3)	27 (40.9)	

Abbreviations: AE, autoimmune encephalitis; ALB, albumin; CNS, central nervous system; CVD, cerebralvascular disease; NCSE, nonconvulsive status encephalitis; RSE, refractory status epilepticus; SD, standard deviation; S‐RSE, super‐refractory status epilepticus.

### 3.2 Logistic analysis and the development of the nomogram

**FIGURE 1:**
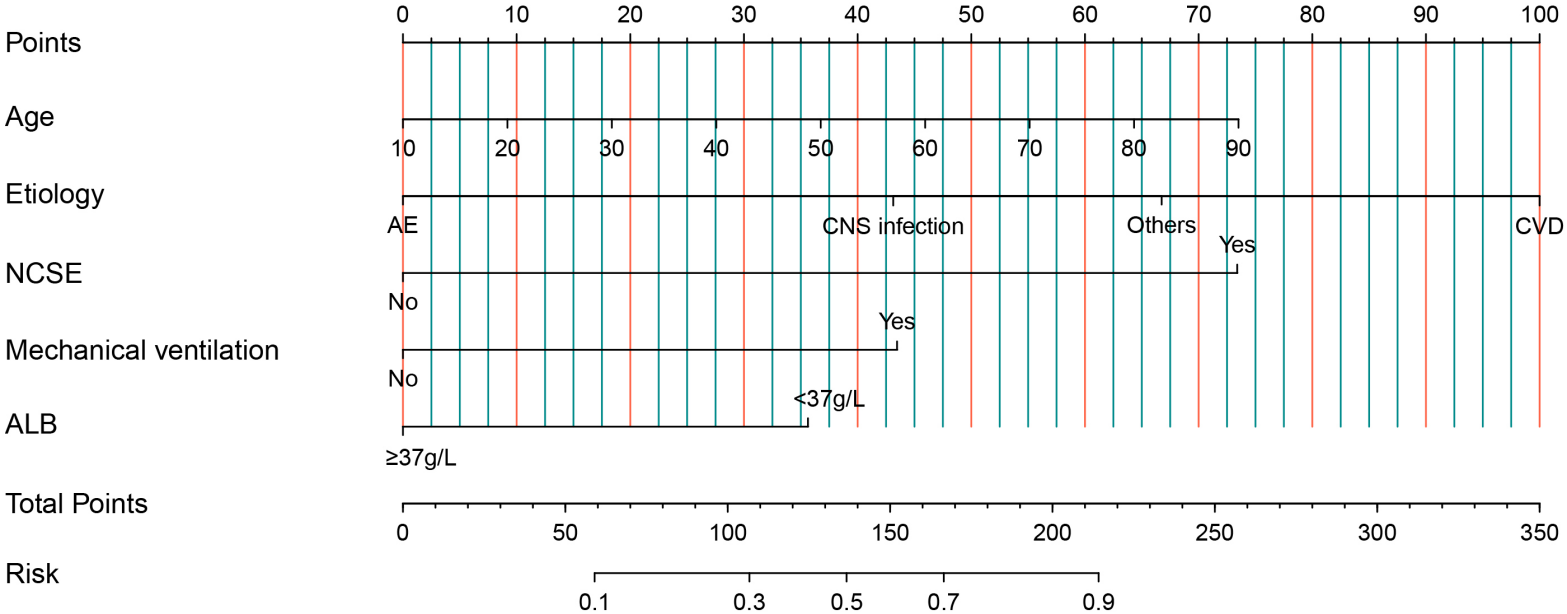
The nomogram for predicting the risk for poor functional outcomes in convulsive status epilepticus. ALB, albumin; CNS, central nervous system; CVD, cerebral vascular disease; NCSE, nonconvulsive status epilepticus.

As shown in Table 2, in univariate analysis, it was found that age, etiology, NCSE, diazepam resistance, the utilization of anesthetics, mechanical ventilation, and abnormal ALB (<37 g/L) at CSE onset were related with the incidence of poor outcome (*p* < 0.1). These variables were further included into multivariate logistic analysis. Following the multivariate logistic analysis, the age, etiology, NCSE, mechanical ventilation, and the abnormal ALB (<37 g/L) at CSE onset were independent risk factors for the poor outcome of CSE patients at 3 months post‐discharge (Table 2). A nomogram containing these five predictors was constructed to estimate the risk for poor prognosis of CSE patients (Figure 1).

**TABLE 2:** Univariate and multivariate logistic regression analysis of factors associated with poor outcome in patients with SE in training cohort.

Variable	Univariate analysis		Multivariate analysis	
	OR (95% CI)	*p* Value	OR (95% CI)	*p* Value
Age (per 1 year)	**1.03 (1.01–1.05)**	**0.002**	**1.03 (1.00–1.06)**	**0.042**
Male	1.17 (0.59–2.34)	0.652		
Etiology				
AE	**Ref**			
CNS infection	**1.52 (0.55–4.25)**	**0.424**	3.15 (0.84–13.25)	0.100
CVD	**9.85 (2.58–49.69)**	**0.002**	**15.68 (2.38–130.38)**	**0.006**
Others	**2.57 (1.06–6.52)**	**0.041**	**6.19 (1.81–24.25)**	**0.006**
Image (%)				
Normal	Ref			
Unilateral lesions	1.67 (0.72–3.94)	0.237		
Bilateral lesions/diffuse cerebral edema	1.93 (0.80–4.76)	0.146		
NCSE	**3.77 (1.81–8.13)**	**0.001**	**7.16 (2.20–26.67)**	**0.002**
Diazepam resistance	**2.43 (1.18–5.12)**	**0.017**	1.71 (0.61–5.01)	0.313
Anesthetics	**2.33 (1.13–4.93)**	**0.024**	0.73 (0.22–2.33)	0.601
Mechanical ventilation	**2.77 (1.37–5.72)**	**0.005**	**3.63 (1.34–10.46)**	**0.013**
ALB < 37 g/L	**3.44 (1.67–7.29)**	**0.001**	**2.76 (1.12–7.03)**	**0.029**

*Note*: The bold values in the univariate logistic analysis refers to variables which were further included into multivariate logistic analysis. The bold values in the multivariate logistic analysis refers to the independent risk factors for the poor outcome of CSE.

Abbreviations: AE, autoimmune encephalitis; ALB, albumin; CNS, central nervous system; CVD, cerebralvascular disease; NCSE, nonconvulsive status encephalitis.

### 3.3 Assessment of the nomogram

**FIGURE 2:**
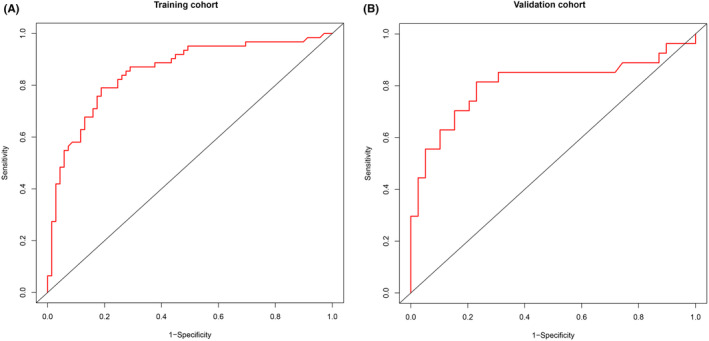
Receiver operating characteristic curves for estimating the risk of poor functional outcomes in the training cohort (A) and validation cohort (B).

**FIGURE 3:**
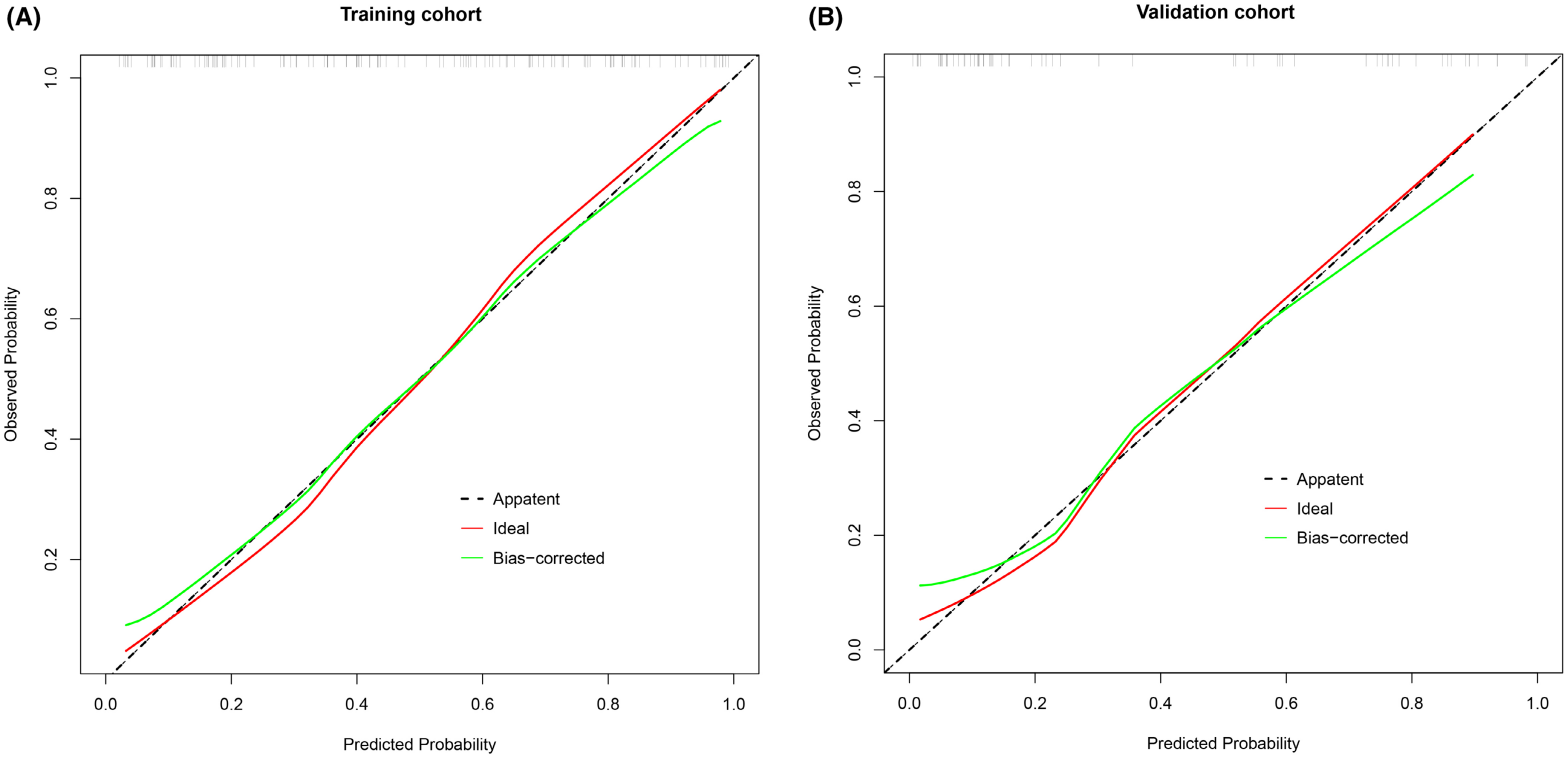
Calibration curves for the nomogram prediction in the training cohort (A) and validation cohort (B).

The C‐index of the nomogram in the training and validation cohorts was 0.853 (95% CI, 0.787–0.920) and 0.806 (95% CI, 0.683–0.923), respectively, which revealed good predictive discrimination. The ROC curves were presented in Figure 2. In addition, the calibration plots also showed a good consistency between the outcomes predicted by the nomogram and the observed outcomes in the cohort at 3 months after discharge (Figure 3).

### 3.4 Website of the nomogram

The nomogram was converted to a web‐based user‐friendly calculator (https://wanjian.shinyapps.io/DynNomapp). This website calculator is freely available online to help clinicians and patients to calculate the risk of poor functional outcomes of patients with CSE at 3 months post‐discharge.

## 4 DISCUSSION

On the basis of our previous study on the END‐IT score,^3^ we modified and internally validated the prediction model to estimate the individualized risk for poor functional outcomes after discharge in CSE patients. The nomogram and web‐based tool we present here, using a combination of demographics, etiology, semiology, complication and biochemical marker, are clinically practical and more precise, which has been a modification of END‐IT score system.

In this prognostic study, the consecutive recruitment lasted for 12 years, which made it a larger database to explore the CSE predictors, compared with our previous study. The age distribution of patients in this prognostic study was closer to that in other study sample from different countries, like the USA and India.^22^, ^23^ To some extent, this overcame the disadvantage that END‐IT derived from a younger SE cohort with a median age of 25. In this study, more senile patients were enrolled. We speculated that it was due to the changes of medical system and policies in China, as well as the economic improvements in northwest China, so that more elderly people can be sent to tertiary hospitals for treatment, whereas in the past, these patients were often reluctant to seek formal treatment. Older age has generally been related to mortality in patients with SE.^18^, ^22^, ^24^ A previous study also showed that the elderly patients had more functional decline than adult patients.^25^ This was because that senile patients were more likely to suffer from SE associated with stroke,^18^ which usually had high mortality and disabled rates. Another possible reason may be that patients with older age were less resistant to complications of SE.^18^ Age was always considered as one of the most clinical determinants for the early prediction of outcomes in SE.^26^ In this study, based on a more representative cohort, we found that age was an independent risk factor for poor prognosis in CSE and was included as a variable of the modified prediction model. In addition, as a continuous variable, age did not need to be categorized in this updated prediction model, which made the predicted result more individual and easier to use.

Etiology was also considered as a determinant factor in the functional outcome of SE.^20^ CNS infection, autoimmune encephalitis and cerebral vascular disease (CVD) are the top three causes of CSE in our 12‐year cohort. Previous studies^27^, ^28^ showed that CNS infection was the primary cause of SE, and encephalitis was associated with markedly poor outcomes in SE especially in developing countries. Therefore, when we proposed END‐IT, only encephalitis is used as an indicator for poor outcomes. However, it should be noted that with accurate identification of etiology of encephalitis, especially the detection of specific antibodies, autoimmune‐related causes become the non‐negligible pathogenic factors of encephalitis in the last decade.^29^ Up to now, autoimmune encephalitis has accounted for at least 20% of all encephalitis cases in adults and become the single most common etiology of new‐onset refractory status epilepticus.^30^, ^31^ Importantly, recent studies reported that the prognosis of a considerable proportion of adult patients with autoimmune encephalitis was good, even if they developed SE during the course of the disease.^31^, ^32^, ^33^, ^34^ Therefore, in this modified prediction model, we singled out autoimmune encephalitis as a separate variable. Besides CNS infection and autoimmune encephalitis, the CVD was another common underlying cause of SE, especially in the elderly.^25^ Since the cerebrovascular disease is somewhat a disability‐causing disease, it seems that the CSE patients whose etiology was CVD, are difficult to get a good functional prognosis. Hence, CVD was also singled out as a separate variable in this modified model.

Nonconvulsive status epilepticus was distinguished from SE with prominent motor phenomena according to the latest definition and classification,^8^ and associated with an increased risk of unfavorable outcomes.^35^ Leitinger et al.^36^ conducted a population‐based study applying this classification of SE 2015 firstly, and found that there was no death in awake patients with NCSE while NCSE patients with disorders of consciousness (DoC) had a higher fatality. The results suggested that the level of consciousness should be determined in researches about NCSE. Here, in our modified model, NCSE refers to NCSE with DoC. We found that NCSE with DoC was an independent risk factor for the poor functional outcome. Increased mortality and neurological deficit rate had been observed with progression from nonrefractory SE to RSE to S‐RSE.^2^, ^37^ To increase operational feasibility, we chose variables to reflect the features of RSE/S‐RSE, including diazepam resistance, the use of anesthetics and mechanical ventilation, as candidate predictors when performing the statistical analysis. These three variables were shown to be associated with a poor outcome in the univariate analysis, but multivariate analysis demonstrated that only mechanical ventilation functioned as an independent predictor. Mechanical ventilation is useful to protect airways and optimize seizure‐related breathing impairment, but as an invasive measure, it also carries risks such as ventilation‐associated pneumonia.^38^ A recent study showed that SE patients supported by mechanical ventilation had less recovery to their premorbid function compared to SE patients without ventilation.^38^ Prolonged mechanical ventilation was also independently associated with an increased risk of death.^19^

The systemic inflammation commonly leads to lower level of serum albumin and may contribute to perpetuate epileptic activity.^17^, ^39^ A previous study found that low levels of albumin at onset were independently associated with RSE and death regardless of seizure severity.^26^ In our study, we demonstrated that the albumin level measured early in CSE was an independent predictor of poor functional outcome, and was chosen as a biochemical indicator in the prediction of the functional outcome.

In our previous work, image was included as an independent predictor in END‐IT. But during clinical practice, we found that neuroimage was not available in each SE patient, which limited the widely use of END‐IT. What's more, the image item in END‐IT referred to the most serious findings during the disease course. A recent study showed that this might not be appropriate, since the remote lesions causing SE could lead to a higher score although they might not necessarily represent negative outcome predictor.^6^ In this study, we modified the definition of the brain lesion. It referred to the brain region generating SE and was identified by comprehensive analysis. We found the responsible brain lesion was not associated with the functional outcome in CSE.

Unlike the previous study which proposed END‐IT, based on the increased sample size, internal validation of this modified prediction model was performed this time, which made the modified prediction model more reliable. This is the first time to develop a nomogram and website calculator to present a prediction model for the outcome in CSE. For healthcare professionals, the nomogram can be used as a reference guide on wards and during consultations.^7^ The widely available website calculator provides a user‐friendly interface in front of a relatively complex statistical model. There are several limitations to this study. First, the information about functional outcome post‐discharge was followed‐up through telephone, which might result in some biases. Second, the prediction model was developed based on data extracted from a cohort from a single university tertiary medical center, not epidemiology derived data. Therefore, modified END‐IT may not be suitable for risk prediction at the population level. In addition, it is inevitable that there was selection bias, potentially making this prediction model more suitable for more severe CSE patients. This may limit the extrapolation of the model to other settings; hence, further multicenter external validation should be performed. Third, our study was retrospective with variability in diagnostic work‐up and management, which may cause information bias, and the response to anti‐SE treatment was not been documented in detail. We plan to conduct a series of prospective studies about SE which will analyze these data. Fourth, pre‐onset mRS and post‐discharge treatments may have impacts on the outcomes of patients with SE, but prior to this study, we did not design a detailed collection of these information.

## 5 CONCLUSIONS

In this prognostic study, as a modification of END‐IT, a nomogram model was developed and converted to a clinically useful tool to estimate the individualized risk for poor functional outcomes after discharge in CSE patients. Further multicenter external validation will be performed in the future.

## AUTHOR CONTRIBUTIONS

Xuan Wang, Fang Yang, and Wen Jiang designed the study. Xuan Wang, Yuan‐Yuan Wang, Qiong Gao, Yao‐Yao Zhang, Chang‐Geng Song, and Jing‐Ya Wei, collected the data. Xuan Wang, Yuan‐Yuan Wang, Jian Wan and Wen Jiang analyzed the data. Chang‐Geng Song and Xiao‐Gang Kang revised the statistical analyses. Xuan Wang wrote the paper. Fang Yang and Wen Jiang revised the paper. All the authors approved the final version of the manuscript.

## FUNDING INFORMATION

This study was supported by National Natural Science Foundation of China (No. 81771406).

## CONFLICT OF INTEREST STATEMENT

The authors declare that they have no conflict of interest.

## PATIENT CONSENT STATEMENT

Informed consent was obtained from the patients or their guardians.

## Supporting information


Figure S1:

## Data Availability

The datasets used and/or analyzed during the current study are available from the corresponding author on reasonable request.
