# Supplementary figures and images for: Development and validation of a nomogram to provide individualized predictions of functional outcomes in patients with convulsive status epilepticus at 3 months: The modified END‐IT tool
Source: CNS Neurosci Ther. 2023 Jun 19;29(12):3935–42. doi: 10.1111/cns.14313 (PMC10651970; doi:10.1111/cns.14313)

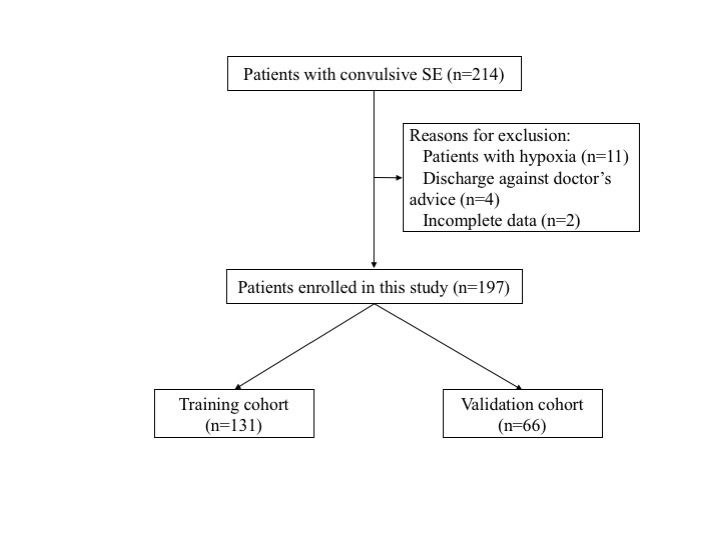

Supplement: Supplementary file 1 — Figure S1: [file CNS-29-3935-s001.jpg]
